# Estimating HIV incidence and number of undiagnosed individuals living with HIV in the European Union/European Economic Area, 2015

**DOI:** 10.2807/1560-7917.ES.2016.21.48.30417

**Published:** 2016-12-01

**Authors:** Anastasia Pharris, Chantal Quinten, Teymur Noori, Andrew J Amato-Gauci, Ard van Sighem, the ECDC HIV/AIDS Surveillance and Dublin Declaration Monitoring Networks

**Affiliations:** 1 European Centre for Disease Prevention and Control (ECDC), Stockholm, Sweden; 2 Stichting HIV Monitoring, Amsterdam, the Netherlands.; 3 Members are listed at the end of the article.

**Keywords:** HIV infection, acquired immunodeficiency syndrome - AIDS, epidemiology, modelling

## Abstract

Since 2011, human immunodeficiency virus (HIV) incidence appears unchanged in the European Union/European Economic Area with between 29,000 and 33,000 new cases reported annually up to 2015. Despite evidence that HIV diagnosis is occurring earlier post-infection, the estimated number of people living with HIV (PLHIV) who were unaware of being infected in 2015 was 122,000, or 15% of all PLHIV (n=810,000). This is concerning as such individuals cannot benefit from highly effective treatment and may unknowingly sustain transmission.

Although preventable through effective public health measures, human immunodeficiency virus (HIV) persists in the 31 countries of the European Union and European Economic Area (EU/EEA) [1]. In this report an analysis of EU/EEA HIV and acquired immunodeficiency syndrome (AIDS) surveillance data from 2015 as well as from prior years is presented. We estimate that, in 2015, 15% (122,000/810,000) of people living with HIV (PLHIV) in the EU/EEA were unaware of their infection.

## Analysis of annual surveillance data

HIV and AIDS surveillance data are reported annually by EU/EEA countries to a joint database for HIV/AIDS within the European Surveillance System (TESSy) coordinated by the European Centre for Disease Prevention and Control (ECDC) and the World Health Organization (WHO) Regional Office for Europe [1]. 

Annual data on HIV diagnoses from 2003 to 2015 were stratified by the presence of a concurrent AIDS diagnosis, i.e. an AIDS-defining event within 3 months of HIV diagnosis, and, for individuals without AIDS, by CD4 cell count (≥ 500, 350–499, 200–349, < 200 cells/mm^3^) at the time of diagnosis [2]. 

The ECDC HIV Modelling Tool version 1.2.2 was used to derive both the estimates of annual HIV incidences, as well as those of the average times from infection to HIV diagnosis each year [3]. These two types of estimates are only presented for the period from 2011 to 2015 due to greater uncertainty of data from the previous years of the study. 

The number of PLHIV in 2015 who were not yet diagnosed was obtained by fitting to data on HIV diagnoses from 2003 to 2011, adjusted for reporting delay, using the ‘Incidence Method’, a CD4 cell count-based back-calculation method [4]. 

Data on the estimated number of diagnosed PLHIV were reported for 2015 by nominated contact points in EU/EEA countries to ECDC as part of the Dublin Declaration monitoring process in 2016 [5]. In the three countries (Iceland, Liechtenstein, and Norway) not reporting estimates of diagnosed PLHIV, data on cumulative HIV cases reported to TESSy through 2015 minus the number of persons reported to have died, were used as a proxy for diagnosed PLHIV. 

The estimated number of diagnosed PLHIV from the Dublin Declaration monitoring reports and the undiagnosed PLHIV estimate from the model were summed to obtain the total number of PLHIV in the EU/EEA for 2015. This was used to derive the proportion undiagnosed PLHIV in that year. 

Comparable estimates of the number of diagnosed PLHIV from the Dublin Declaration monitoring are not available for earlier years than 2015, thus the estimates of PLHIV overall and of the proportion of PLHIV unaware of their infection could only be calculated for 2015. 

## New yearly diagnoses of HIV and estimated annual HIV incidences

In 2015, 29,727 cases of HIV were diagnosed and reported in the EU/EEA, resulting in a rate of 6.3 per 100,000 population when adjusted for reporting delay. The notification rate and the number of new HIV diagnoses reported have remained unchanged since 2011, with between 29,000 and 33,000 new cases reported annually (notification rates of between 6.3 and 6.5 per 100,000 population) [1]. 

**Figure 1:**
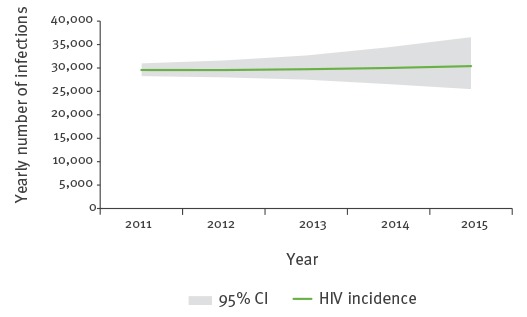
Estimated human immunodeficiency virus incidence by year, European Union/European Economic Area, 2011–2015

CI: confidence interval; HIV: human immunodeficiency virus.

HIV incidence estimates present a stable trend similar to that of HIV cases notified via the surveillance system, with an estimated 30,000 new infections (95% confidence interval (CI): 25,000–37,000) for the year 2015 (Figure 1).

## Evolution of CD4 cell count at diagnosis and of the delay between infection and diagnosis in years up to 2015

Late diagnosis is a persistent issue in EU/EEA countries. In the 24 EU/EEA countries reporting data on CD4 cell count at diagnosis among 18,103 persons >15 years-old diagnosed in 2015, nearly half (n=8,490; 47%) of all cases had a CD4 cell count of less than 350 cells/mm^3^, while 28% (n=5,094) had advanced HIV infection (CD4 < 200 cells/mm^3^). In the thirteen countries reporting the CD4 cell count consistently over time, the median CD4 cell count at diagnosis increased significantly from 314 cells/mm^3^ in 2005 to 377 cells/mm^3^ in 2015 (p < 0.001). 

**Figure 2:**
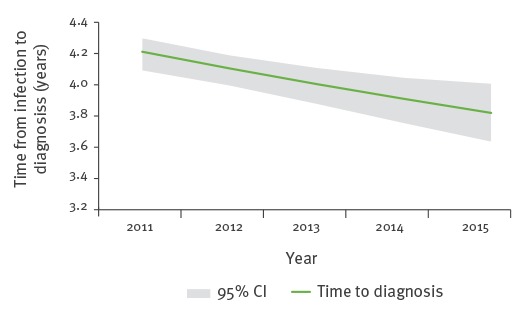
Average time from infection to diagnosis of human immunodeficiency virus by year, European Union/European Economic Area, 2011–2015

Meanwhile, the estimated expected time from HIV infection to diagnosis decreased from 4.2 years (95% CI: 4.1–4.3) on average in 2011 to 3.8 years (95% CI: 3.6–4.0) in 2015 (Figure 2).

CI: confidence interval.

## Estimated number of persons living with undiagnosed infection

The number of people living with undiagnosed HIV in the EU/EEA in 2015 was estimated at 122,000 (95% CI: 111,000–136,000). The total estimated number of PLHIV in the EU/EEA was 810,000 (0.2% of adult population ≥15 years-old). The resulting estimated proportion of those living with undiagnosed HIV was 15% (95% CI: 14–17%).

## Background and discussion 

The Joint United Nations Programme on HIV/AIDS (UNAIDS) has set forth ambitious global targets to end AIDS by 2030 and established ‘90–90–90’ targets for 2020 (90% of all people living with HIV will know their status; 90% of people aware of their status will receive sustained antiretroviral treatment; and 90% of those on antiretroviral treatment will have viral suppression) [6]. To better understand HIV trends and estimate the status of the first target (90% of people living with HIV aware of their status) in the EU/EEA, we analysed HIV and AIDS surveillance data through 2015. Despite high treatment coverage [7], earlier diagnosis, and concerted prevention efforts, there is no decline in the number of HIV diagnoses or the number of HIV infections in the EU/EEA in recent years.

This analysis shows that the estimated proportion of all PLHIV in the EU/EEA who are living with undiagnosed HIV is 15%. Using a similar CD4 back-calculation approach on surveillance data, it was estimated that 16% of PLHIV in the United States in 2013 were undiagnosed [8]. The estimate presented here for the EU/EEA is considerably lower than the previous estimate of 30%, which is based on data from 2005 [9]. This could be a result of several factors. First, this might be a reflection of increased or more targeted testing as supported by the observed increase in the CD4 cell counts at diagnosis and decreased time from HIV infection to diagnosis. With treatment guidelines moving towards earlier treatment, and growing awareness of the benefits of early antiretroviral treatment, more persons at higher risk of infection may get tested more frequently. Second, the annual number of new infections is approximately the same as the number of new diagnoses. Thus the number living with undiagnosed HIV remains relatively stable and as people on treatment live longer with HIV, the proportion of undiagnosed persons with HIV will naturally become smaller in relation to the ever-increasing population of diagnosed PLHIV [1]. Third, new methods to estimate the undiagnosed fraction are available and these are informed by improved surveillance data.

While approximately 85% of those living with HIV in the EU/EEA are estimated to be diagnosed, it remains to be seen whether it is possible for the EU/EEA to reach the UNAIDS first ‘90’ target by 2020. A more appropriate measure to gauge progress may be to monitor the reduction in the number of undiagnosed individuals living with HIV, rather than monitoring a proportion where the denominator is steadily increasing.

The average time between HIV infection and diagnosis, while improving, is still nearly four years. As starting antiretroviral treatment earlier reduces morbidity and mortality among HIV-positive individuals [10] and reduces HIV transmission to HIV-negative partners [11] it is essential that individuals are diagnosed early. In order to further reduce the time from HIV infection to diagnosis, countries should consider implementing and scaling up innovative approaches to promote greater access to and uptake of HIV testing by those most at risk, including community-based testing, self-testing and home sampling, as well as indicator-condition-guided testing.

This pooled EU/EEA estimate conceals differences between key populations, where the trend over time and proportion undiagnosed is likely to vary. An EU estimate is also more heavily weighted towards the situation of countries with larger populations. The proportion of persons remaining undiagnosed is diverse across countries that have carried out national analyses [12-17] and is likely to be significantly higher than 15% in many countries and among some key population groups. In Europe, further work is needed to carry out key population-specific and country-level estimates of HIV incidence and the undiagnosed number in a standardised manner in order to more accurately monitor progress and inform testing programmes.

This analysis has several important limitations. It was not possible to adjust the data for countries that did not have full coverage of HIV surveillance prior to 2012 (such as Italy and Spain) and this may have resulted in an underestimation of PLHIV. Conversely, PLHIV may have been overestimated due to the inability of many countries to fully link their death, emigration and surveillance registries and, thus, accurately measure the number of those diagnosed still living with HIV. For these reasons, it was not possible to obtain a reliable estimate of PLHIV using only HIV notification data reported to TESSy. Instead, data reported by countries through the Dublin Declaration monitoring process on people diagnosed and living with HIV were used, and these were obtained using different methods, with some countries unable to completely remove all cases who had died or emigrated from the number diagnosed. Until approaches to estimate diagnosed PLHIV can be further standardised, country-reported data provide the best current estimate in the EU/EEA.

## Conclusions

Overall, this analysis demonstrates that recent HIV incidence is constant in the EU/EEA, and that a substantial number of people are living with undiagnosed HIV. Efforts to obtain better national and key population-specific estimates and to further increase the offer and uptake of HIV testing among those most at risk remain key to informing HIV prevention efforts and achieving global targets to reduce HIV incidence and the number of persons remaining undiagnosed in the EU/EEA.

## Notes

**Members of the ECDC HIV/AIDS Surveillance and Dublin Declaration Monitoring Networks:** ECDC HIV/AIDS Surveillance and Dublin Declaration Monitoring Networks include: Austria: Daniela Schmid, Irene Rueckerl, Robert Zangerle; Belgium: Andre Sasse, Dominique Van Beckhoven, Frédéric Denauw; Bulgaria: Tonka Varleva, Vyara Georgieva; Croatia: Tatjana Nemeth Blazic, Jasmina Pavlic, Josip Begovac; Cyprus: Maria Koliou, Linos Hadjihannas, Anna Demetriou; Czech Republic: Marek Maly, Veronika Šikolová; Denmark: Susan Cowan, Jan Fouchard; Estonia: Kristi Rüütel, Anna-Liisa Pääsukene; Finland: Kirsi Liitsola, Mika Salminen, Henrikki Brummer- Korvenkontio; France: Françoise Cazein, Josiane Pillonel, Florence Lot, Jean- Christophe Comboroure; Germany: Barbara Gunsenheimer-Bartmeyer, Matthias an der Heiden, Gesa Kupfer, Ulrich Marcus; Greece: Georgios Nikolopoulos, Dimitra Paraskeva, Vasileia Konte; Hungary: Maria Dudas, Katalin Szalay; Iceland: Haraldur Briem, Gudrun Sigmundsdottir; Ireland: Derval Igoe, Kate O’Donnell, Caroline Hurley, Fiona Lyons; Italy: Barbara Suligoi, Anna Caraglia; Latvia: Šarlote Konova; Liechtenstein: Sabine Erne; Lithuania: Irma Čaplinskienė; Luxembourg: Aurelie Fischer, Patrick Hoffmann; Malta: Jackie Maistre Melillo, Tanya Melillo; Netherlands: Eline Op de Coul, Silke David; Norway: Hans Blystad, Arild Johan Myrberg; Poland: Magdalena Rosinska, Iwona Wawer; Portugal: Kamal Mansinho, Helena Cortes Martins, Teresa Melo; Romania: Mariana Mardarescu; Slovakia: Peter Truska, Jan Mikas; Slovenia: Irena Klavs; Spain: Asuncion Diaz, Oliver Nuñez, Olivia Castillo; Sweden: Maria Axelsson, Anders Sönnerborg; United Kingdom: Peter Kirwan, Cuong Chau, Sandra Okala, Alison Brown, Valerie Delpech.
